# Difference between the blood samples of patients with bone and joint tuberculosis and patients with tuberculosis studied using machine learning

**DOI:** 10.3389/fsurg.2022.1031105

**Published:** 2023-01-06

**Authors:** Zhen Ye, Jichong Zhu, Chong Liu, Qing Lu, Shaofeng Wu, Chenxing Zhou, Tuo Liang, Jie Jiang, Hao Li, Tianyou Chen, Jiarui Chen, Guobing Deng, Yuanlin Yao, Shian Liao, Chaojie Yu, Xuhua Sun, Liyi Chen, Hao Guo, Wuhua Chen, Wenyong Jiang, Binguang Fan, Xiang Tao, Zhenwei Yang, Wenfei Gu, Yihan Wang, Xinli Zhan

**Affiliations:** First Affiliated Hospital of Guangxi Medical University, Nanning, China

**Keywords:** tuberculosis, bone tuberculosis, joint tuberculosis, machine learning algorithms, prediction model, nomogram

## Abstract

**Background:**

Tuberculosis (TB) is a chronic infectious disease. Bone and joint TB is a common type of extrapulmonary TB and often occurs secondary to TB infection. In this study, we aimed to find the difference in the blood examination results of patients with bone and joint TB and patients with TB by using machine learning (ML) and establish a diagnostic model to help clinicians better diagnose the disease and allow patients to receive timely treatment.

**Methods:**

A total of 1,667 patients were finally enrolled in the study. Patients were randomly assigned to the training and validation cohorts. The training cohort included 1,268 patients: 158 patients with bone and joint TB and 1,110 patients with TB. The validation cohort included 399 patients: 48 patients with bone and joint TB and 351 patients with TB. We used three ML methods, namely logistic regression, LASSO regression, and random forest, to screen the differential variables, obtained the most representative variables by intersection to construct the prediction model, and verified the performance of the proposed prediction model in the validation group.

**Results:**

The results revealed a great difference in the blood examination results of patients with bone and joint TB and those with TB. Infectious markers such as hs-CRP, ESR, WBC, and NEUT were increased in patients with bone and joint TB. Patients with bone and joint TB were found to have higher liver function burden and poorer nutritional status. The factors screened using ML were PDW, LYM, AST/ALT, BUN, and Na, and the nomogram diagnostic model was constructed using these five factors. In the training cohort, the area under the curve (AUC) value of the model was 0.71182, and the C value was 0.712. In the validation cohort, the AUC value of the model was 0.6435779, and the C value was 0.644.

**Conclusion:**

We used ML methods to screen out the blood-specific factors—PDW, LYM, AST/ALT, BUN, and Na^+^—of bone and joint TB and constructed a diagnostic model to help clinicians better diagnose the disease in the future.

## Introduction

Tuberculosis (TB) is a chronic infectious disease mainly caused by *Mycobacterium tuberculosis* (1). The main transmission route of TB is respiratory tract transmission. The prevalence of TB in China is approximately 66/100,000 in sputum smear-positive patients and approximately 119/100,000 in sputum smear-negative patients (2). TB tends to occur in people with low immunity, such as patients with diabetes, infants, patients with AIDS, and immunosuppressant users (3). Common symptoms include cough, sputum, hemoptysis, and dyspnea (4). Severe complications can lead to cardiopulmonary failure and even death.

Extrapulmonary TB is reported in 14% of TB cases (5). Bone and joint TB is an infectious disease caused by tuberculosis bacilli through blood, lymph, or direct spread to bone tissue, most of which are secondary to TB. Bone and joint TB tends to occur in the spine, hip joint, and knee joint, often presenting with pain in the joints (6). Bone and joint TB is diagnosed by blood examination, imaging, and pathological biopsy. Commonly used treatment methods include drug therapy and surgery (7).

Machine learning (ML) is the science of how computers learn from data. ML has its roots in computer science and is a subfield of artificial intelligence. It is a cross between statistics, which deals with learning relationships from data, and computer science, which focuses on efficient algorithms. ML is extensively used in clinical data processing (8, 9).

Numerous studies have been conducted on TB, but few studies have focused on the differences between patients with bone and joint TB and patients with TB. The gold standard for the diagnosis of bone and joint TB is pathological results but many primary hospitals lack imaging tools and pathological examination and can thus judge bone and joint TB only by symptoms and blood examination results. In addition, bone and joint TB is often difficult to get TB infected tissues in minimally invasive puncture diagnosis, which easily affects the clinician's judgment of the condition. In this study, we collected the clinical data of patients with bone and joint TB and common TB to determine the differences by using ML methods to help clinicians better diagnose and treat bone and joint TB.

## Patients and methods

### Patients

This study was voluntary, and all patients signed an informed consent form. This study was approved by the Ethics Committee of the First Affiliated Hospital of Guangxi Medical University (Supplementary Materials 2) [No. 2022-KY-E-(287)].

From 2012 to 2022, we collected a total of 12,770 hospitalized patients diagnosed with TB in the First Affiliated Hospital of Guangxi Medical University. The inclusion criteria for patients with pulmonary TB were as follows: (1) *Mycobacterium tuberculosis* detected in sputum smears or lesions and diagnosed as TB by two or more specialists. (2) Complete erythrocyte sedimentation rate (ESR) examination, blood routine examination, liver function examination, renal function examination, high sensitivity C-reactive protein (hs-CRP), and plasma electrolyte examination data were available for the patient. (3) Patients voluntarily participated in the study. In the case of minors, their guardians signed on their behalf. (4) The patient exhibited good compliance and no serious cardiovascular and cerebrovascular diseases. The inclusion criteria for patients with bone and joint TB were as follows: (1) Patients were hospitalized in the Department of Spinal Bone Disease, the First Affiliated Hospital of Guangxi Medical University, and diagnosed as bone and joint TB by surgery or biopsy. (2) Complete erythrocyte sedimentation rate examination, blood routine examination, liver function examination, renal function examination, and hs-CRP and plasma electrolyte examination data were available for the patient. (3) Patients voluntarily participated in the study. In the case of minors, their guardians signed on their behalf. (4) The patient had good compliance and no serious cardiovascular and cerebrovascular diseases.

The exclusion criteria were as follows: (1) Patients who did not have *Mycobacterium tuberculosis* in sputum smears or lesions. (2) Patients with missing clinical data. (3) Patients with serious cardiovascular and cerebrovascular diseases. (4) Patients who did not agree to participate in the study.

A total of 1,667 patients were enrolled in the study. Among them, 206 patients were diagnosed with bone and joint TB, and 1,461 patients were diagnosed with common TB. Among the patients diagnosed with bone and joint TB, eight were hip TB, 14 were knee TB, eight were cervical TB, 63 were lumbar TB, two were thoracolumbar TB, and 111 were thoracic TB. We randomly assigned patients to the training and validation cohorts. There were 1,268 patients in the training cohort: 158 patients with bone and joint TB and 1,110 patients with TB. The validation cohort included 399 patients: 48 patients with bone and joint TB and 351 patients with TB.

All data were collected from the information system of the First Affiliated Hospital of Guangxi Medical University, and the patients' ID numbers were used for the search. Blood routine examination included white blood cell (WBC) count, red blood cell (RBC) count, hematocrit value (HCT), mean corpuscular volume (MCV), mean corpuscular hemoglobin (MCH), mean corpuscular hemoglobin concentration (MCHC), Platelet hematocrit (PLT), platelet distribution width (PDW), neutrophil percentage (NEUT), lymphocytes percentage (LYM), monocyte percentage (MO), eosinophils percentage (EO), RBC volume distributing width (CV), and thrombocytocrit (PCT). Liver and kidney function examination included total bilirubin (TBil), direct bilirubin (DBil), indirect bilirubin (IBil), total protein (TP), albumin (ALB), globulin (GLB), gamma-glutamyl transpeptidase (GGT), total bile acid (TBA), aspartate aminotransferase (AST), alanine aminotransferase (ALT), A alkaline phosphatase (ALP), prealbumin (PAB), cholinesterase (ChE), blood urea nitrogen (BUN), creatinine (Cr), uric acid (UA), bicarbonate radical (HCO), creatinine clearance rate (Ccr), and Cysteine C (Cys-C). Plasma electrolyte examination included K^+^, Na^+^, Cl^−^, Ca^2+^, and Mg^2+^. The data of all screened patients were complete, but hs-CRP was not tested in some patients, and hs-CRP was statistically analyzed separately.

## Statistical analysis

IBM SPSS Statistics 23 and R software (version 4.1.3; https://www.R-project.org) were used for data analysis and visualization. Student's *t*-test was used to compare the means of continuous variables between the two cohorts (patients with bone and joint TB and patients with TB) (10). The *t*-test data were normally distributed with uniform variance (11). Hs-CRP and gender differences were tested by chi-square test (12). Multiple checks were performed on the calculations to ensure that they were correct. A two-sided probability of less than 0.05 was considered statistically significant for all analyzed data (13, 14).

Univariate and multivariate logistic regression were analyzed and visualized using the “rms,” “glmnet,” and “plyr” R packages. LASSO regression was analyzed and visualized using the “glmnet” package (15). The random forest was analyzed and visualized using the “randomForest” package. The nomogram construction and C value calculation were performed using the “rms” package (16). The area under the curve (AUC) of the ROC curve and Harrell's concordance index were used to evaluate the performance of nomogram predictions (17). Harrell's concordance index (C-index) was calculated to assess nomogram discrimination by using a bootstrap method with 1,000 samples (18). The “corrplot” package was used to analyze the correlation of the independent variables. The “rma” and “rmda” packages were used to calculate the net benefits and visualize the decision curve (19).

### Random forest

Random forest is a learning machine based on decision trees and can be used to predict continuous variables and improve prediction accuracy (20, 21). An increase in mean squared (%IncMSE) and an increase in node purity (IncNodePurity) are two methods of random forest for prediction. %IncMSE is a random assignment of each predictor variable. If this variable is more important, the prediction error will be larger if it is randomly replaced. IncNodePurity is measured by the sum of squared residuals. In both cases, the larger the value, the more important the prediction variable. Both can be regarded as important indicators of predictive variables (22).

## Results

Tables 1–3 list the differences in ESR, blood routine examination, liver function examination, renal function examination, and plasma electrolytes between patients with bone and joint TB and patients with TB. In the ESR and blood routine examination (Table 1), ESR was significantly increased in patients with TB, but patients with bone and joint TB had higher values than patients with TB. The RBC, HCT, PDW, and LYM of patients with bone and joint TB were lower than those of patients with TB. NEUT and CV of patients with bone and joint TB were higher than those of patients with pulmonary TB.

**Table 1:** The differences ESR and blood routine examination.

	Training cohort			Validation cohort		
Type	Bone and joint tuberculosis	Tuberculosis	*P*-value	Bone and joint tuberculosis	Tuberculosis	*P*-value
	(*N* = 158)	(*N* = 1110)		(*N* = 48)	(*N* = 351)	
**Sex**						
Male	108	753	0.896	33	232	0.715
Female	50	357		15	119	
**Age**						
Mean (SD)	56.92 (17.2)	55.73 (18.6)	0.450	60.42 (17.44)	56.08 (17.8)	0.113
**ESR**						
Mean (SD)	51.67 (31.5)	44.83 (30.8)	0.009	56.91 (31.29)	46.9 (30.9)	0.036
**WBC**						
Mean (SD)	8.99 (3.8)	8.37 (4.85)	0.121	9.69 (9.25)	8.22 (3.56)	0.051
**RBC**						
Mean (SD)	3.8 (0.83)	4.08 (0.78)	<0.001	3.68 (4.10)	4.10 (0.78)	0.001
**HCT**						
Mean (SD)	0.31 (0.056)	0.34 (0.06)	<0.001	0.31 (0.054)	0.34 (0.06)	<0.001
**MCV**						
Mean (SD)	83.48 (8.49)	84.56 (9.79)	0.189	84.16 (8.75)	84.85 (9.91)	0.645
**MCH**						
Mean (SD)	27.3 (3.43)	27.69 (4.1)	0.213	27.6 (3.28)	27.80 (3.78)	0.709
**MCHC**						
Mean (SD)	325.87 (12.6)	326.53 (18.3)	0.657	327.34 (10.42)	326.89 (12.04)	0.805
**PLT**						
Mean (SD)	329.7 (143.2)	309.1 (135.1)	0.077	307.5 (121.2)	322.9 (138.5)	0.464
**PDW**						
Mean (SD)	0.153 (0.03)	0.165 (0.007)	<0.001	0.154 (0.03)	0.1423 (0.03)	0.017
**NEUT**						
Mean (SD)	0.71 (0.13)	0.65 (0.13)	<0.001	0.70 (0.12)	0.66 (0.13)	0.029
**LYM**						
Mean (SD)	0.162 (0.09)	0.21 (0.10)	<0.001	0.165 (0.09)	0.204 (0.1)	0.010
**MO**						
Mean (SD)	0.086 (0.035)	0.092 (0.040)	0.059	0.090 (0.036)	0.093 (0.037)	0.594
**EO**						
Mean (SD)	0.037 (0.05)	0.04 (0.04)	0.455	0.038 (0.039)	0.038 (0.04)	0.965
**CV**						
Mean (SD)	0.166 (0.089)	0.158 (0.031)	0.004	0.170 (0.04)	0.15 (0.03)	<0.001
**PCT**						
Mean (SD)	0.26 (0.104)	0.24 (0.095)	0.031	0.25 (0.09)	0.27 (0.11)	0.093

The red text means that the *p* value was <0.05. SD, standard deviation. ESR, erythrocyte sedimentation rate; WBC, White blood cell count; RBC, Red blood cell count; HCT, hematocrit value; MCV, Mean corpuscular volume; MCH, Mean corpuscular hemoglobin; MCHC, Mean corpuscular hemoglobin concentration; PLT, Platelet hematocrit; PDW, Platelet distribution width; NEUT, neutrophil percentage; LYM, lymphocytes percentage; MO, Monocyte percentage; EO, eosinophils percentage; CV, RBC volume distributing width; PCT, thrombocytocrit.

**Table 2:** The differences liver function examination, renal function examination and plasma electrolyte examination.

	Training cohort			Validation cohort		
Type	Bone and joint tuberculosis	Tuberculosis	*P*-value	Bone and joint tuberculosis	Tuberculosis	*P*-value
	(*N* = 158)	(*N* = 1110)		(*N* = 48)	(*N* = 351)	
**TBil**						
Mean (SD)	14.56 (25.9)	9.78 (11.6)	<0.001	11.2 (7.10)	7.99 (5.51)	<0.001
**DBil**						
Mean (SD)	8.06 (18.7)	4.69 (8.1)	<0.001	5.79 (4.45)	3.96 (3.94)	0.003
**IBil**						
Mean (SD)	6.59 (8.22)	5.09 (4.27)	<0.001	5.32 (3.73)	3.96 (3.95)	0.004
**DBil/TBil**						
Mean (SD)	0.48 (0.15)	0.44 (0.15)	0.001	0.51 (0.15)	4.03 (2.77)	0.612
**TP**						
Mean (SD)	64.9 (7.42)	65.9 (8.15)	0.148	8.27 (1.19)	7.48 (0.40)	0.006
**ALB**						
Mean (SD)	32.8 (4.74)	34.96 (5.8)	<0.001	32.8 (4.03)	35.87 (5.31)	<0.001
**GLB**						
Mean (SD)	32.23 (7.47)	30.96 (7.44)	0.044	30.2 (7.92)	30.23 (6.10)	0.964
**ALB/GLB**						
Mean (SD)	1.08 (0.34)	1.20 (0.37)	<0.001	1.13 (0.30)	1.24 (0.32)	0.03
**GGT**						
Mean (SD)	66.24 (52.8)	68.43 (73.6)	0.718	56.03 (37.65)	67.02 (79.5)	0.347
**TBA**						
Mean (SD)	13.75 (33)	11.07 (22.62)	0.192	7.58 (9.38)	8.67 (12.5)	0.563
**AST**						
Mean (SD)	29.62 (18.32)	29.5 (44.1)	0.974	37.17 (64.24)	28.45 (27.16)	0.094
**ALT**						
Mean (SD)	21.77 (17.42)	25.62 (42.9)	0.265	24.0 (40.32)	24.1 (21.87)	0.979
**AST/ALT**						
Mean (SD)	1.75 (1.09)	1.42 (0.84)	<0.001	1.65 (1.12)	1.50 (1.43)	0.476
**ALP**						
Mean (SD)	107.8 (49.75)	102.34 (66.65)	0.321	94.15 (33.11)	102.43 (81.0)	0.484
**PAB**						
Mean (SD)	139.7 (69.2)	171.1 (73.5)	<0.001	0.170 (0.04)	0.15 (0.03)	0.002
**BUN**						
Mean (SD)	5.31 (4.83)	4.57 (3.45)	0.018	5.34 (4.48)	4.75 (2.83)	0.22
**Cr**						
Mean (SD)	83.44 (105.66)	76.04 (62.45)	0.209	74.27 (49.8)	82.54 (83.9)	0.505
**UA**						
Mean (SD)	308.08 (167.81)	350.36 (194.84)	0.010	293.65 (167.5)	345.74 (180.66)	0.06
**HCO**						
Mean (SD)	25.1 (4.21)	25.1 (3.79)	0.976	25.83 (4.82)	25.78 (4.23)	0.943
**Ccr**						
Mean (SD)	83.19 (30.16)	88.07 (30.58)	0.061	85.0 (33.27)	97.8 (35.45)	0.018
**Cys-C**						
Mean (SD	1.10 (0.64)	1.02 (0.7)	0.200	1.12 (0.77)	0.94 (0.61)	0.057
**K^+^**						
Mean (SD)	4.02 (0.47)	4.0 (0.49)	0.721	3.98 (0.36)	3.95 (0.52)	0.693
**Na^+^**						
Mean (SD)	137.25 (3.79)	138.67 (3.94)	<0.001	138.0 (3.94)	138.5 (0.365)	0.462
**Cl^−^**						
Mean (SD)	101.86 (4.45)	102.87 (4.58)	0.009	101.9 (4.56)	100.65 (4.20)	0.052
**Ca^2+^**						
Mean (SD)	2.16 (0.16)	2.18 (0.16)	0.101	2.14 (0.17)	2.18 (0.17)	0.05
**Mg^2+^**						
Mean (SD)	0.80 (0.12)	0.86 (0.14)	<0.001	0.797 (0.105)	0.872 (0.121)	<0.001

The red text means that the *p* value was <0.05. SD, standard deviation. TBil, total bilirubin; DBil, direct bilirubin; IBil, indirect bilirubin; TP, total protein; ALB, albumin; GLB, globulin; GGT, gamma-glutamyl transpeptidase; TBA, Total bile acid; AST, aspartate aminotransferase; ALT, alanine aminotransferase; ALP, A alkaline phosphatase; PAB, prealbumin; ChE, cholinesterase; BUN, Blood urea nitrogen; Cr, creatinine; UA, Uric acid; HCO, bicarbonate radical; Ccr, Creatinine clearance rate; Cys-C, Cysteine C.

**Table 3:** The differences hs-CRP examination.

	Training cohort			Validation cohort		
Type	Bone and joint tuberculosis	Tuberculosis	*P*-value	Bone and joint tuberculosis	Tuberculosis	*P*-value
	(*N* = 125)	(*N* = 1110)		(*N* = 38)	(*N* = 351)	
<10	29 (23.2%)	442 (39.8%)	<0.001	5 (13.2%)	148 (42.2%)	0.001
>=10	96 (76.8%)	668 (60.2%)		33 (86.8%)	203 (57.8%)	

The red text means that the *P* value was <0.05. hs-CRP, High sensitivity C-reactive protein.

In the liver function examination (Table 2), TBil, DBil, IBil, DBil/IBil, GLB, and AST/ALT in patients with bone and joint TB were higher than those in patients with TB, and ALB, ALB/GLB, and PAB in patients with bone and joint TB were lower than those in patients with TB. In the renal function examination (Table 2), UA was lower, and BUN was higher in patients with bone and joint TB than in those with TB. Ccr was lower in patients with bone and joint TB in both training and validation cohorts, but there was no significant difference in the training cohort. In the examination of plasma electrolytes (Table 2), Na^+^, Cl, and Mg in patients with bone and joint TB were lower than those in patients with TB, and the differences were statistically significant. As can be seen in Table 3, the proportion of hs-CRP ≥ 10 was significantly higher in patients with bone and joint TB than in patients with TB.

**Figure 1:**
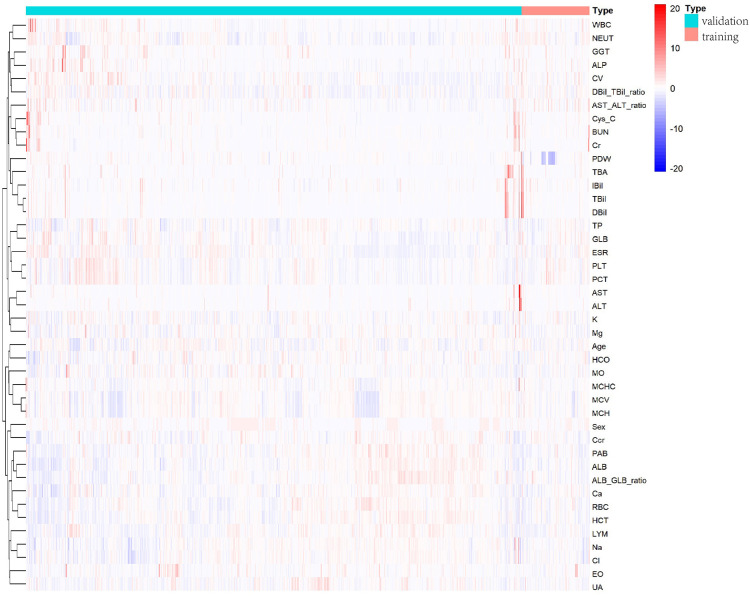
Bone and joint TB and TB training cohorts and validation cohorts heat maps.

**Figure 2:**
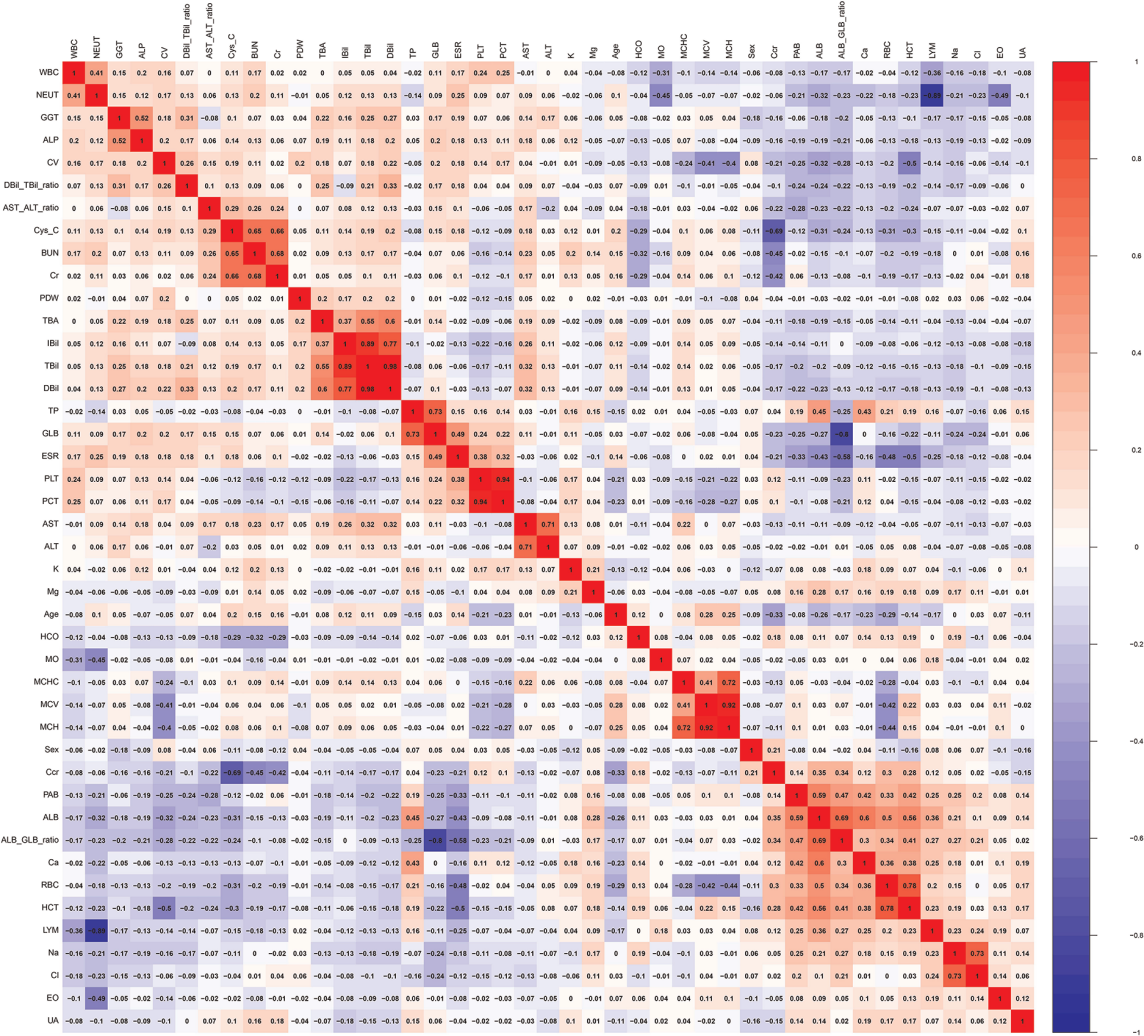
Correlation between all variables.

The heat map (Figure 1) shows the distribution of all the variables in the training and validation cohorts. Figure 2 illustrates the relationship between all the variables. Positive correlations were noted between BUN and Cys-C, Cr and BUN, TBil and IBil, DBil and TBil, DBil and IBil, GLB and TB, PCT and PLT, ALT and AST, MCH and MCHC, MCH and MCV, ALB/GLB and ALB, HCT and RBC, and Cl^−^ and Na^+^. a clear negative correlation was observed between LYM and NEUT, Ccr and Cys-C, and ALB/GLB and GLB.

**Figure 3:**
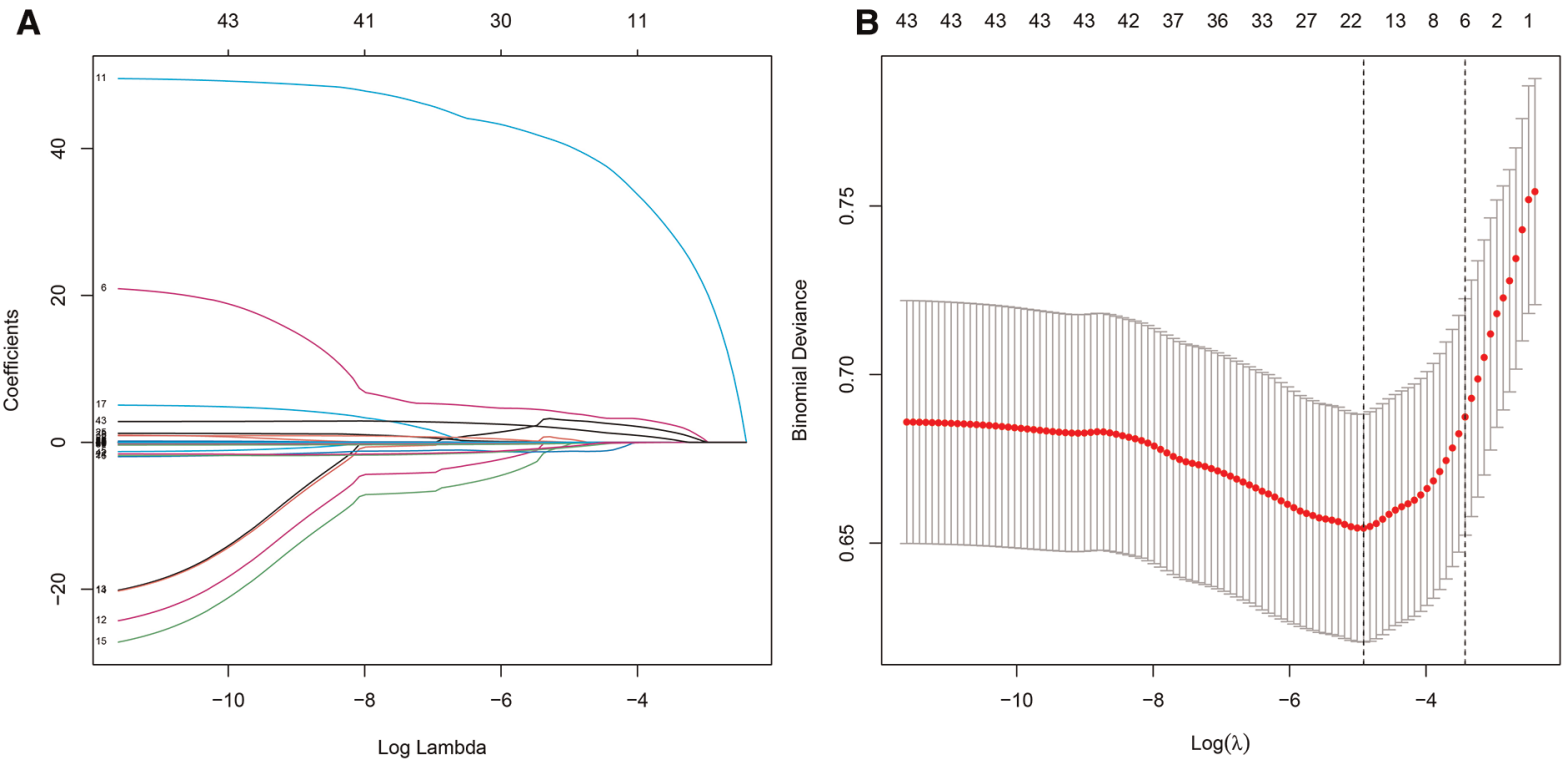
LASSO regression. (**A**) Results of LASSO regression for all variables. (**B**) There were significant differences in 17 factors between bone and joint TB and TB.

We screened the variables by using univariate and multivariate logistic regression, Lasso regression, and random forest to obtain the factors with the most obvious differences between the two cohorts. Table 4 presents the results of univariate regression and multivariate regression. Figure 3A shows the results of Lasso regression for all the variables. As can be seen from Figure 3B, the best effect was achieved when 19 factors were selected to be included in the Lasso regression model; Table 5 lists the 19 variables included in the Lasso regression results.

**Table 4:** Univariate logistic regression and multivariate logistic regression predicted bone tuberculosis.

Type	Univariate OR (95% CI)	*P*-value	Multivariate OR (95% CI)	*P*-value
Age	0.9965 (0.9873–1.0055)	0.450	/	/
ALB	1.0641 (1.034–1.0947)	<0.001	0.9210 (0.8575–0.9893)	0.0241
ALB/GLB	2.5574 (1.5724–4.2222)	0.0002	2.8507 (0.5749–14.1353)	0.1997
ALP	0.9988 (0.9967–1.0013)	0.3232	/	/
ALT	1.0050 (0.9987–1.0148)	0.2437	/	/
AST	0.9999 (0.9967–1.0052)	0.9736	/	/
AST/ALT	0.7277 (0.6246–0.8488)	<0.001	0.8341 (0.6887–1.0103)	0.0636
BUN	0.9595 (0.9269–0.9964)	0.0227	0.9698 (0.9182–1.0243)	0.2719
Ca	2.3550 (0.8407–6.5216)	0.1009	/	/
Ccr	1.0054 (0.9998–1.0858)	0.0607	0.9999 (0.9925–1.0074)	0.9789
Cl	1.0482 (1.0115–1.0858)	0.0092	0.97789 (0.9209–1.0384)	0.4656
Cr	0.9988 (0.9970–1.0010)	0.2234	/	/
CV	0.0010 (0.00001–0.1244)	0.0040	0.2172 (0.0002–282.726)	0.6765
Cys_C	0.8789 (0.7251–0.9917)	0.2074	/	/
DBil	0.9793 (0.9662–0.9917)	0.0015	0.8154 (0.5165–1.2874)	0.3811
DBil/TBil	0.1789 (0.0630–0.5114)	0.0013	0.3233 (0.0610–0.1847)	0.1847
EO	5.0283 (0.1035–483.49)	0.4545	/	/
ESR	0.9932 (0.9880–0.9984)	0.0097	1.0036 (0.9952–1.0120)	0.4051
GGT	1.0004 (0.9982–1.0031)	0.7181	/	/
GLB	0.9788 (0.9588–0.9999)	0.0450	1.0207 (0.9666–1.0779)	0.4603
HCO	0.9993 (0.9565–1.0433)	0.9756	/	/
HCT	1875.70 (124.71 –29,129.04)	<0.001	5447.49 (10.1422–292,592.7)	0.0073
IBil	0.9581 (0.9327–0.9838)	0.0014	0.7664 (0.4870–0.2502)	0.2502
K	0.9397 (0.6709–1.3260)	0.7206	/	/
LYM	237.97 (35.73–1725.94)	<0.001	164.9533 (1.2679–0.0398)	0.0398
MCH	1.0275 (0.9852–1.0719)	0.2102	/	/
MCHC	1.0024 (0.9933–1.0140)	0.6562	/	/
MCV	1.0113 (0.9943–1.0283)	0.1886	/	/
Mg	28.9297 (7.4546–116.75)	<0.001	13.7496 (2.8109–67.256)	0.0012
MO	90.2245 (0.9762–10,355.38)	0.0574	52.8651 (0.0973–28,721.44)	0.2169
Na^+^	1.0896 (1.0467–1.1340)	<0.001	1.0232 (0.9545–1.0968)	0.5176
NEUT	0.0255 (0.0063–0.0993)	<0.001	4.6243 (0.0962–222.31)	0.4383
PAB	1.0066 (1.0040–1.0093)	<0.001	1.0025 (0.9989–1.0061)	0.1715
PCT	0.16798 (0.0337–0.8800)	0.0318	145.246 (0.2563–82,313.954)	0.1238
PDW	1.054 × 10^19^ (2.7267 × 10^14^–1.4052 × 10^24^)	<0.001	4.3985 × 10^21^ (5.4315 × 10^15^–3.5619 × 10^27^)	<0.001
PLT	0.9990 (0.9978–1.0001)	0.0771	0.9961 (0.9917–1.005)	0.0855
RBC	1.5783 (1.2730–1.9625)	<0.001	0.7185 (0.4649–1.1102)	0.1364
Sex	1.0241 (0.7196–1.4749)	0.8965	/	/
TBA	0.9963 (0.9910–1.0026)	0.2010	/	/
TBil	0.9851 (0.9758–0.9938)	0.0011	1.2349 (0.7852–1.9421)	0.36108
TP	1.0155 (0.9947–1.0369)	0.1475	/	/
UA	1.0013 (1.0003–1.0023)	0.0099	1.0012 (1.0001–1.0023)	0.0397
WBC	0.9774 (0.9495–1.0089)	0.1292	/	/

The red text means that the *p* value was <0.05. ESR, erythrocyte sedimentation rate; WBC, White blood cell count; RBC, Red blood cell count; HCT, hematocrit value; MCV, Mean corpuscular volume; MCH, Mean corpuscular hemoglobin; MCHC, Mean corpuscular hemoglobin concentration; PLT, Platelet hematocrit; PDW, Platelet distribution width; NEUT, neutrophil percentage; LYM, lymphocytes percentage; MO, Monocyte percentage; EO, eosinophils percentage; CV, RBC volume distributing width; PCT, thrombocytocrit;TBil, total bilirubin; DBil, direct bilirubin; IBil, indirect bilirubin; TP, total protein; ALB, albumin; GLB, globulin; GGT, gamma-glutamyl transpeptidase; TBA, Total bile acid; AST, aspartate aminotransferase; ALT, alanine aminotransferase; ALP, A alkaline phosphatase; PAB, prealbumin; ChE, cholinesterase; BUN, Blood urea nitrogen; Cr, creatinine; UA, Uric acid; HCO, bicarbonate radical; Ccr, Creatinine clearance rate; Cys-C, Cysteine C.

**Table 5:** Nineteen variables screened by lasso regression.

PDW	LYM	MO	EO	CV
IBil	DBil/TBil	GGT	AST/ALT	PAB
BUN	UA	HCO	K	Na^+^
Ca	Mg			

**Figure 4:**
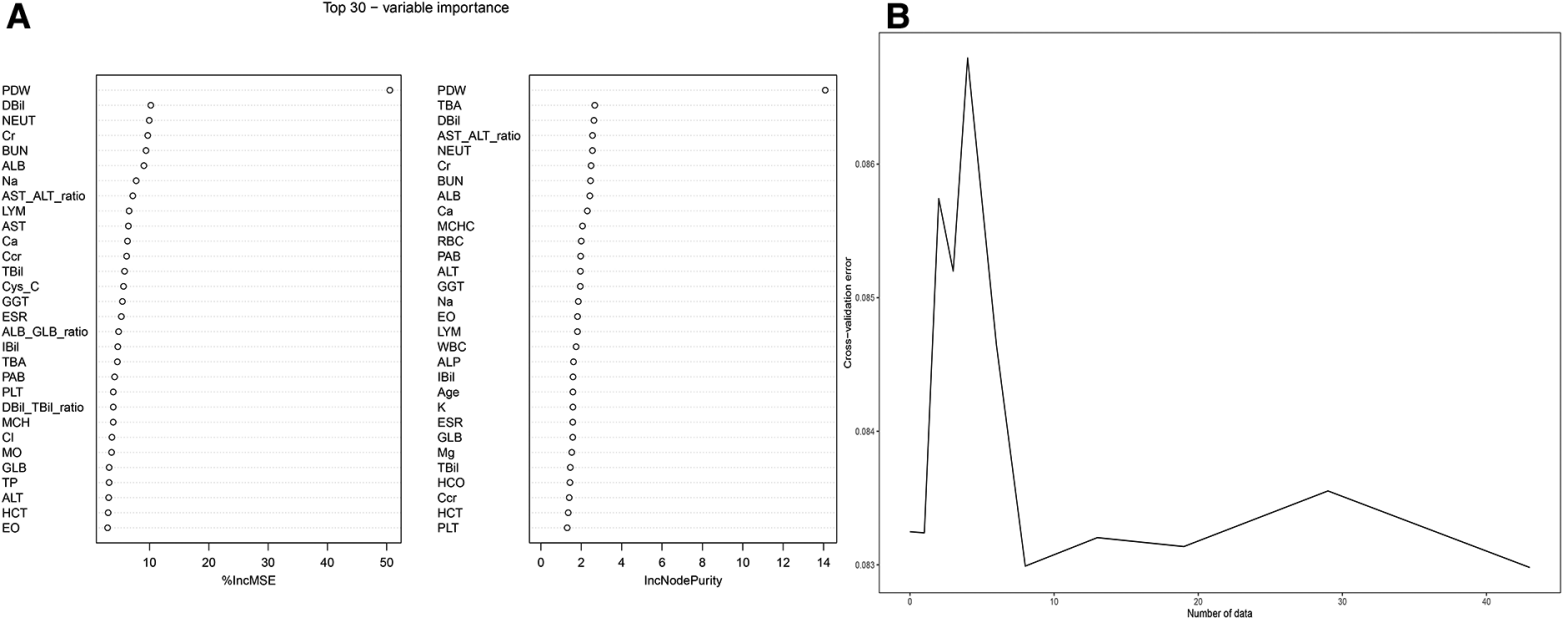
Random forest. (**A**) %IncMSE and IncNodePurity respectively screened the top 30 important factors. (**B**) The best diagnostic model was obtained when random forest included 18 factors.

**Figure 5:**
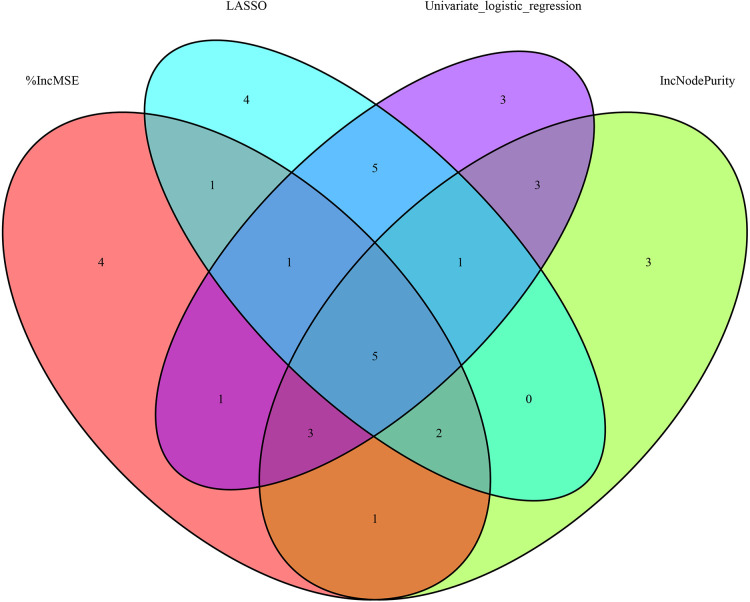
4 methods venn diagram.

Figure 4A depicts the 30 most important variables screened by the two calculation methods of %IncMSE and IncNodePurity. The effect was better when 9–18 factors were screened out in the random forest and included in the model (Figure 4B). Finally, we used %IncMSE and IncNodePurity to screen out the first 18 important variables and combined them with the factors screened by univariate logistic regression and the 19 factors screened by Lasso regression to screen out five factors as the final model (Figure 5). The final five factors screened were PDW, LYM, AST/ALT, BUN, and Na^+^.

**Figure 6:**
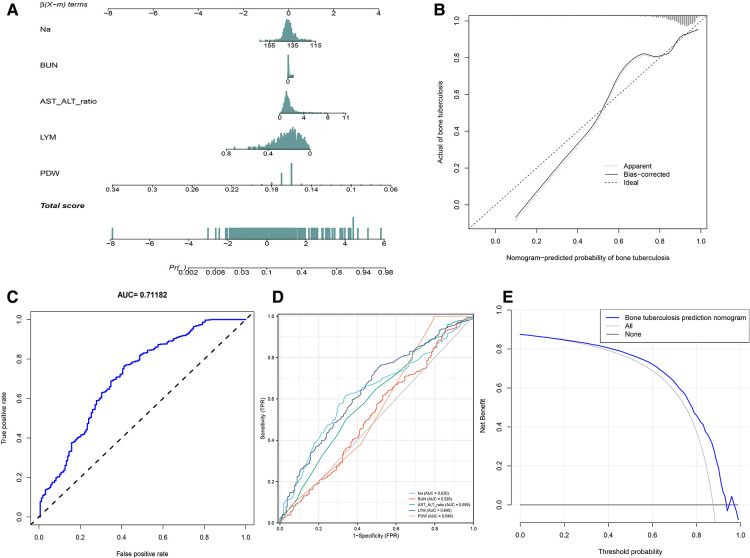
Diagnosis model. (**A**) Nomogram to predict the probability of bone and joint TB. (**B**) Calibration curves for predicting bone and joint TB. (**C**) The AUC value of the prediction model. (**D**) The AUC values of Na^+^, BUN, AST/ALT, LYM, and PDW. (**E**) Decision curve analysis for the prediction model.

**Figure 7:**
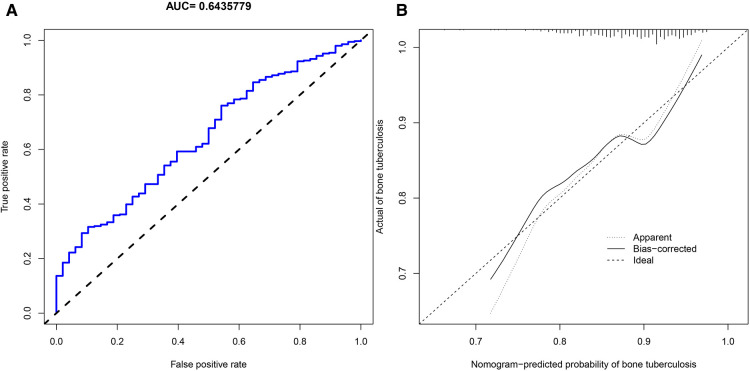
Validation cohort. (**A**) AUC values of the diagnostic model of bone and joint TB in the validation cohort. (**B**) Calibration curves for predicting bone and joint TB in the validation cohort.

We applied PDW, LYM, AST/ALT, BUN, and Na^+^ to construct the nomogram diagnostic model (Figure 6A). The nomogram exhibited a good fitting degree (Figure 6B). The AUC value of the diagnostic model was 0.71182 (Figure 6C). A decision curve was constructed to analyze the clinical utility of the model; the model exhibited clinical utility when the threshold of the model was in the range of 3%–100%, and the NONE line of the decision curve was above the ALL line (Figure 6E). The AUC value obtained by placing the diagnostic model in the validation cohort was 0.6435779 (Figure 7A), and a good fit was obtained in the validation cohort (Figure 7B).

## Discussion

The aim of this study was to explore the differences between patients with bone and joint TB and patients with TB to screen out the most representative factors through the application of ML in the clinical data of the two groups. It is hoped that in the future, it will help clinicians to diagnose bone and joint TB earlier and more easily, and help patients get earlier treatment (23).

TB is a chronic inflammatory reaction caused by *Mycobacterium tuberculosis*. The specific response of humans to *Mycobacterium tuberculosis* is called “tuberculosis granuloma.” It is a nodular mass that mixes inflammatory cells and immune cells in the local collection (5). “Granulomatous inflammation” is used to describe a mononuclear infiltration of macrophages and lymphocytes (24). Tissue injury and inflammatory stimulation cause elevated hs-CRP and ESR levels. hs-CRP and ESR can be used as activity indicators to monitor diseases (25, 26). As can be seen in Table 1, the average values of these two factors are much greater in patients with TB than in normal controls. This is the same as observed in the study by Li et al. (27). WBC and NEUT are commonly used as indicators of bacterial infection in clinical practice (28). The mean values of hs-CRP, ESR, WBC, and NEUT in patients with TB were lower than those in patients with bone and joint TB, indicating that patients with bone and joint TB were more seriously infected.

PDW is a common indicator in routine blood examination; hemodilution or some blood-related diseases can lead to a decrease in PDW. In the PDW control study of TB and normal subjects by Xu et al., the mean PDW of TB subjects was lower than that of normal subjects (29). LYM is a type of WBC, originally derived from bone marrow hematopoietic stem cells, which differentiate into cells with different functions (30). Lymphocytes play an important role in adaptive immunity, enabling the immune system to recognize and remember antigens. LYM reduction is common in infections, TB, immune system diseases, and the use of hormones (31). LYM in patients with TB is lower than that in normal subjects (32).

We found that the mean PDW and LYM values of patients with bone and joint TB on routine blood tests were significantly lower than those of patients with TB. The lower mean value of LYM in patients with bone and joint TB indicates that such patients have lower immune capacity and are at higher levels of inflammation (30). This issue is also illustrated by the inverse relationship between LYM and NEUT inflammatory markers in Figure 2. Lymphocytes are divided into T lymphocytes, B lymphocytes, and NK cells. Different lymphocytes have different characteristics. Lymphocytopenia in patients with bone and joint TB is more obvious and needs further study. PLT was elevated in patients with bone and joint TB (Table 1), and the average value was higher than normal. PLT is an inflammatory marker and is elevated in patients with TB (33). PLT and PDW exhibited a negative correlation (Figure 2). We believe that the relative increase in PLT in patients with bone and joint TB leads to a decrease in PDW.

Bilirubin is a pigment made from hemoglobin in red blood cells. When hemoglobin dies, it breaks down to biliverdin, which in response to NADPH and H ions generates bilirubin (34). In the liver function test, TBil, DBil, and IBil of patients with bone and joint TB were higher than those of patients with TB, indicating a correlation between aging erythrocytes in patients with bone and joint TB. The decrease in RBC in patients with bone and joint TB proved our view (Table 1). However, AST/ALT was increased in patients with bone and joint TB, mainly due to the decrease in ALT. ALT is a sensitive indicator of liver damage (35). The higher average value of ALT in patients with TB indicates that patients with pulmonary TB may have a greater liver function burden. Antituberculous drugs also affect liver function, and the reason for the increase in ALT needs to be further investigated. ALB is commonly used as a measure of nutritional status, and TB is a wasting disease, which is confirmed by the fact that ALB was lower than normal in both groups (36). The average ALB of patients with bone and joint TB was lower than that in patients with TB, which may be related to the longer and more severe infection, leading to more decline in nutritional status. The increase in GLB is usually due to the invasion of viruses and bacteria. The immune system produces increased GLB against viruses and bacteria, which also indicates that patients with bone and joint TB are more severely attacked by *Mycobacterium tuberculosis* (37).

Furthermore, BUN increased, and UA decreased in patients with bone and joint TB.

BUN and Cr are both waste products of human metabolism. The kidney is also the only organ that clears Cys-C. In clinical practice, the increase of BUN, Cys-Cand Cr is often used to indicate abnormal renal function, so there is a positive correlation between them (38). BUN is the product of the catabolism of human protein; 90% of BUN needs to be excreted by the kidney (39). Protein breakdown in patients with bone and joint TB is greater than that in patients with TB, and the nutrient consumption is greater. UA content is usually proportional to the intake of purine content, and there was no difference in the diet between the two groups; Thus, only the endogenous reduction of purine production in UA can be considered. Currently, it is not known how UA works, and further research is required. In the examination of plasma electrolytes, Na^+^, Cl^−^, and Mg^2+^ were slightly decreased in patients with bone and joint TB, but there is no clear implication in clinical practice.

We used univariate logistic regression, LASSO regression, and random forest to screen the clinical data of 206 patients with bone and joint TB and 1,461 patients with TB to identify the five most variable factors. Finally, we constructed the nomogram diagnostic model by using five factors, namely PDW, LYM, AST/ALT, BUN, and Na^+^, and achieved relatively good efficiency. The proposed diagnostic model will help clinicians more easily diagnose patients with bone and joint TB, prevent the progression of TB in time, and give patients more timely treatment. Especially in the face of patients with atypical bone and joint TB, many doctors are afraid to use anti-TB drugs without clear pathological results. Our diagnostic model can help doctors to a certain extent. Of course, a more accurate diagnosis of bone and joint TB still requires the imaging and pathological results of TB and the experience of clinicians.

Few blood investigation studies on patients with bone and joint TB have been conducted. We used ML methods to screen patients' ESR, blood routine, liver and kidney function, and plasma electrolytes, which is a novel feature of our study. We used ML to process big data, screen out specific factors, and construct diagnostic models. However, our study also has some limitations. For example, the data used in the study belonged to a single hospital. The use of multicenter data will provide more convincing results. In addition, cases of bone and joint TB are relatively rare. The number gap between bone and joint TB and TB patients is also large, which can also cause errors in the accuracy of the model. The diagnostic performance of the nomogram is not good enough. This study is only a preliminary exploration of the blood characteristics of patients with bone and joint TB, and there are many directions for further research.

## Conclusion

We used ML to screen out the blood-specific factors PDW, LYM, AST/ALT, BUN, and Na^+^ of bone and joint TB and constructed a diagnostic model to help clinicians better diagnose the disease in the future.

## Data Availability

The original contributions presented in the study are included in the article/**Supplementary Material**, further inquiries can be directed to the corresponding author/s.
